# Engagement with patients’ sexual problems: a comparative study among general practitioners and obstetrician-gynecologists

**DOI:** 10.1097/GME.0000000000002551

**Published:** 2025-05-06

**Authors:** Anna Aromaa, Katja Kero, Sanna-Mari Manninen, Tero Vahlberg, Päivi Polo-Kantola

**Affiliations:** 1 Department of Obstetrics and Gynecology, Satasairaala Central Hospital, the Wellbeing Services County of Satakunta, Pori; 2 Department of Obstetrics and Gynecology, University of Turku; 3 Department of Obstetrics and Gynecology, Turku University Hospital, Turku; 4 Department of Health Promotion, Metropolia University of Applied Sciences, Helsinki; 5 Department of Biostatistics, University of Turku and Turku University Hospital, Turku, Finland

**Keywords:** Attitude, General practitioner, Obstetrician-gynecologist, Practice pattern, Questionnaire, Sexual problem

## Abstract

**Objectives:**

Even though good sexual health is an important part of well-being, the evaluation of patients’ sexual problems is not necessarily routine for physicians. We compared engagement (attitudes, barriers to bringing up, and practice patterns) with patients’ sexual problems among general practitioners (GPs) and obstetrician-gynecologists (OB/GYNs) with special regard for the sex and age of the physician.

**Methods:**

A web-based questionnaire was used for data collection from 2 samples of physicians. The completed questionnaires from 402 GPs and 299 OB/GYNs were eligible for analysis. In the statistical analysis, the GPs were compared with the OB/GYNs as entire groups with multivariable binary logistic regression adjusted for sex and age. In addition, interaction and subgroup analysis by sex and age groups were both carried out.

**Results:**

Both GPs and OB/GYNs considered treating sexual problems to be an important health care practice. However, compared with the OB/GYNs, the GPs were less likely to inquire about sexual problems during general medical history-taking [adjusted odds ratio (aOR): 0.23, 95% CI: 0.16-0.33, *P* < 0.0001] and more likely to consider diagnosing female sexual problems as being difficult (aOR: 2.44, 95% CI: 1.73-3.44, *P* < 0.0001). Compared with the OB/GYNs, the GPs were more likely to report having barriers—for example, “shortness of the appointment time” (aOR: 2.36, 95% CI: 1.53-3.63, *P* < 0.0001), “personal attitudes and beliefs” (aOR: 2.07, 95% CI: 1.41-3.67, *P* = 0.001), and “lack of knowledge about sexual medicine” (aOR: 2.05, 95% CI: 1.36-3.10, *P* = 0.001).

**Conclusions:**

Both GPs and OB/GYNs considered the treatment of sexual problems to be an important health care practice; however, the engagement with patients’ sexual problems among GPs was less structured.

Sexual health is often ignored or passed over for other health care problems. Asking about patients’ sexual problems within general history taking is not usually routine for physicians.^1^,^2^,^3^ Nevertheless, both physicians^1^,^3^,^4^,^5^ and patients^6,7^ regard sexual health as essential. According to patients, physicians infrequently assess sexual issues,^8,9^ yet most patients agree that health care personnel should inquire regularly about sexual health.^4,6,10^ Both obstetrician-gynecologists (OB/GYNs)^11^ and general practitioners (GPs)^12,13^ are often the first physicians to be contacted regarding sexual concerns. According to a U.S. study among women seeking help for sexual problems, a plurality, 42%, consulted an OB/GYN, and the second most, 24%, consulted a GP.^14^

Studies evaluating the engagement with sexual problems with regard to physician specialty are limited. In a U.S. study with 383 patients, GPs were reported to ask about sexual health less frequently than OB/GYNs.^6^ In another U.S. study among 416 physicians (family practice, internal medicine, OB/GYN, pediatrics, and others), GPs reported a lower frequency of taking a sexual history compared with OB/GYNs.^15^ Further, in a U.S. study among 257 GPs and 248 OB/GYNs, GPs reported more barriers to initiating dialogs about sexual health.^16^ Previous studies show sex and age differences in attitudes and practice patterns in sexual medicine. Some studies,^2,15,17^ but not all,^3,13,18^ found female physicians to be more active in assessing sexual issues. Likewise, younger OB/GYNs have been shown to be more active in sexual history taking.^2,17^ However, one previous study among GPs showed no such age-related difference.^13^

Given this context, in the present study, we compared attitudes, barriers, and practice patterns regarding the engagement with patients’ sexual problems by GPs and OB/GYNs with special regard to the sex and age of the physicians. We hypothesized that the management of sexual problems is more routine for OB/GYNs, as obstetrics and gynecology encompass hormones, fertility, and reproductive organs, which are highly relevant to sexuality. The results of our study can be used to enhance and allocate education on sexual medicine.

## METHODS

### Participants and data collection

This study was part of the Finnish Sexual Medicine Education study, which investigated practice patterns and levels of education in sexual medicine in Finland.

**FIG. 1:**
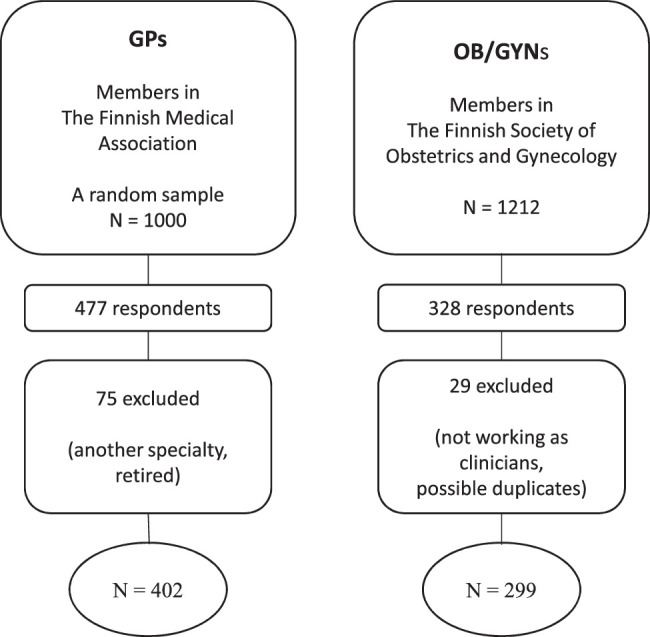
Flowchart of the study. GP, general practitioner; OB/GYN, obstetrician-gynecologist.

Detailed data collection methods are described in our previous articles.^19,20^ The responses from the GPs and the OB/GYNs were compared. The GP participants were a random sample of GPs who were current members of the Finnish Medical Association and indicated that a municipal health center was their primary workplace. In accordance with the Finnish Medical Association’s policy, contact information was restricted to 1,000 Finnish GPs. Of the GP respondents, 75 were excluded because they reported not being among the target group (they were retired or in another specialty). The OB/GYN participants were recruited through the registry of the Finnish Society of Obstetrics and Gynecology. Of the 1,212 OB/GYN respondents, 29 were excluded because they reported not being among the target group (not working as clinicians) or possibly double-answered. The completed questionnaires from 402 GPs and 299 OB/GYNs were eligible for analysis (Fig. 1). Basic characteristics of the respondents are shown in Table 1. For statistical analysis, respondents were divided by sex (female; male) and age (young, <40 y; middle-aged, 40-49 y; and late middle-aged, ≥50 y).

**TABLE 1:** Basic characteristics

	GPs (N = 402)	OB/GYNs (N = 299)
Age (Y): mean (SD)	45.0 (10.7)	47.1 (11.0)
Age (Y): range	27-65	28-74
Age (Y)	n (%)	n (%)
Young (<40)	147 (36)	82 (27)
Middle-aged (40-49)	111 (28)	107 (36)
Late middle-aged (≥50)	144 (36)	110 (37)
Sex		
Female	302 (75)*a*	278 (93)*b*
Male	100 (25)	21 (7)

GP, general practitioner; OB/GYN, obstetrician-gynecologist.

^*a*^ Corresponding to the sex distribution of Finnish GPs in municipal health centers (65% female).^21^

^*b*^ Corresponding to the sex distribution of Finnish OB/GYN specialists (87% female).^22^

### Questionnaires

The study questionnaires were adapted from the Portuguese SEXOS study questionnaire^23,24^ by permission, and also described in our previous studies,^19,20^ including the following 2 fields:

A) Attitudes and practice patterns in the treatment of sexual problems (4 items).

Each item was rated on a 5-point scale defined as follows: 1 = “totally disagree,” 2 = “disagree,” 3 = “agree,” 4 = “totally agree,” and 5 = “cannot say.”

1. Treating sexual problems is an important health care practice.
2. Diagnosing female sexual problems is difficult.
3. I often inquire about sexual problems during general medical history taking.
4. My organization has specific instructions for where to refer patients with sexual problems for continued care. And 2 separate questions:
  - When taking a patient’s sexual history, do you ask how satisfied the patient is with their sexual life? “Always”/“Usually”/“Seldom”/“Never.”
  - How do you usually conduct sexual history taking? (You can choose more than one option). “Open conversation”/“Structured interview”/“A questionnaire”/“I do not take a sexual history.”

B) Barriers to bringing up sexual problems.

Each item was rated on a 5-point scale defined as follows: 1 = “not at all,” 2 = “some,” 3 = “much,” 4 = “very much,” and 5 = “cannot say.”

Bringing up sexual problems with patients is hindered by:

1. Shortness of the appointment time.
2. Sexual problem not being a priority in the appointment.
3. Personal attitudes and beliefs.
4. Personal discomfort when addressing sexual problems.
5. Lack of knowledge about sexual medicine.
6. Lack of experience with sexual medicine.
7. Lack of effective treatment for sexual problems.
8. Fear of failing to respond to patients’ sexual problems.
9. Disability of the patient.

The web-based questionnaires were programmed to not proceed in case of a missing answer, ensuring that every submitted questionnaire was complete.

### Statistical analysis

The data are described using frequencies (percentages). In the analyses, each item in fields A and B was dichotomized [A (items 1-4): “totally agree” or “agree” vs “totally disagree” or “disagree”; and B: “very much” or ”much” vs ”not at all” or ”some”]. Question 5 in field A was dichotomized as “always” or “usually” versus “never” or “seldom.” “Cannot say” responses in field A in items 1-4 and in field B were omitted from the analyses. Question 6 in field A was a multiple-choice question.

First, GPs and OB/GYNs were compared as entire groups. In the 2 fields of interest (A and B), multivariable binary logistic regression was carried out with adjustment for sex (female/male) and age (<40/40-49/≥50 y). For both fields, each question was examined separately. Second, interaction analysis was carried out to investigate whether the associations of specialty (GPs vs OB/GYNs) on the outcomes were different between sex or age groups. Finally, GPs were compared with OB/GYNs in the subgroups of sex and age. The results are presented as adjusted odds ratios (aORs) with 95% CIs. *P* values <0.05 were considered statistically significant. Statistical analyses were performed using the SAS System for Windows, version 9.4 (SAS Institute Inc.).

### Ethical approval

The Sexual Medicine Education study respects the Declaration of Helsinki in terms of ensuring the participants' anonymity and obtaining their informed consent. The study protocol was reviewed and approved by the ethics committee of the University of Turku (44/2017). Replying to the questionnaire implied consent, which was made clear to the respondents in the introduction of the questionnaire.

## RESULTS

### Attitudes and practice patterns in the treatment of sexual problems

**FIG. 2:**
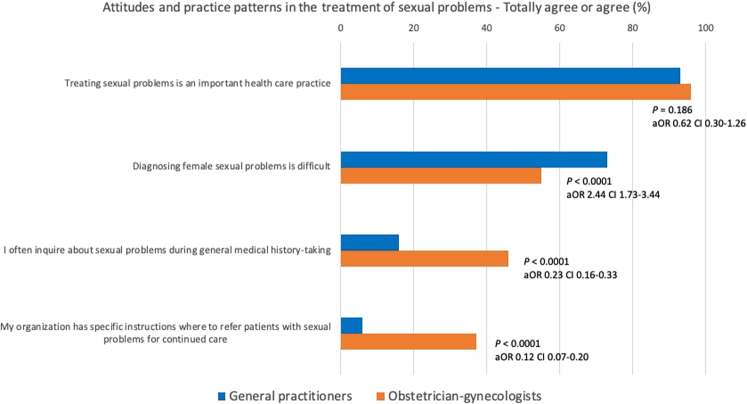
Attitudes and practice patterns in the treatment of sexual problems. aOR, adjusted odds ratio; CI, confidence interval.

The results showing attitudes and practice patterns in the treatment of sexual problems are presented in Figure 2 and Table 2. Both GPs and OB/GYNs considered treating sexual problems to be an important health care practice, with no differences according to sex or age. However, compared with OB/GYNs, GPs were less likely to inquire about sexual problems during general medical history taking. In diagnosing female sexual problems, GPs were more likely to consider diagnosing as difficult. Furthermore, GPs were less likely to report that their organization had specific instructions concerning patient referral to continued care. There were no significant interactions either between sex and specialty or age group and specialty (data not shown). The same differences between GPs and OB/GYNs were found in all subanalyses, except in one age group: No difference was found between young GPs and young OB/GYNs in reporting the difficulty of diagnosing female sexual problems.

**TABLE 2:** Attitudes and practice patterns in the treatment of sexual problems

	Totally agree or agree			
	aOR (95% CI)			
	GP (N/total) vs OB/GYN (N/total)			
	Treating sexual problems is an important health care practice	Diagnosing female sexual problems is difficult	I often inquire about sexual problems during general medical history taking	My organization has specific instructions where to refer patients with sexual problems for continued care
Female (*P*)*a*	0.227	0.0001	0.0001	0.0001
GP vs OB/GYN	0.62 (0.29-1.35)	2.25 (1.56-3.26)	0.24 (0.16-0.35)	0.12 (0.07-0.21)
	283/301 vs 265/276	207/278 vs 149/262	48/299 vs 124/277	18/286 vs 94/258
Male (*P*)*a*	0.468	0.001	0.001	0.0003
GP vs OB/GYN	0.45 (0.05-3.87)	6.10 (2.04-18.28)	0.17 (0.06-0.48)	0.07 (0.02-0.29)
	20/21 vs 90/100	60/87 vs 6/21	17/98 vs 12/21	6/92 vs 8/20
Age <40 (*P*)*b*	0.241	0.185	<0.0001	<0.0001
GP vs OB/GYN	0.39 (0.08-1.88)	1.54 (0.81-2.90)	0.16 (0.08-0.34)	0.10 (0.04-0.30)
	138/147 vs 78/80	97/130 vs 50/76	13/146 vs 31/82	5/137 vs 20/76
Age 40-49 (*P*)*b*	0.295	0.001	<0.0001	<0.0001
GP vs OB/GYN	0.53 (0.17-1.73)	3.06 (1.54-6.06)	0.26 (0.13-0.51)	0.11 (0.05-0.27)
	102/111 vs 102/107	75/100 vs 58/100	20/108 vs 47/106	10/107 vs 43/102
Age ≥50 (*P*)*b*	0.825	<0.0001	<0.0001	<0.0001
GP vs OB/GYN	0.88 (0.27-2.81)	3.15 (1.84-5.41)	0.25 (0.15-0.44)	0.12 (0.05-0.26)
	135/143 vs 105/110	95/135 vs 47/107	32/143 vs 58/110	9/134 vs 39/100

The “cannot say” responses were omitted from the analyses. In each question the responses “totally agree” or “agree” / the number of analyzed responses are shown in the lower column.

aOR >1 indicates higher agreement with the statement.

aOR <1 indicates lower agreement with the statement.

aOR, adjusted odds ratio; GP, general practitioner; OB/GYN, obstetrician-gynecologist.

^*a*^ The multivariable binary logistic regression was carried out with adjustment of age (<40/40-49/≥50 y).

^*b*^ The multivariable binary logistic regression was carried out with adjustment of sex (female/male).

As for asking about sexual life satisfaction, compared with the OB/GYNs, GPs were less likely to report it (aOR: 0.53, 95% CI: 0.38-0.92, *P* < 0.0001). There were no interactions between sex and specialty (*P* = 0.502). However, an interaction between age group and specialty was found (*P* = 0.006). Compared with late middle-aged OB/GYNs, late middle-aged GPs were less likely to ask about satisfaction (aOR: 0.29, 95% CI: 0.17-0.49, *P* < 0.0001). No difference was found between GPs and OB/GYNs in methods of sexual history taking; nor were there differences in subanalyses by sex or age groups or interactions (data not shown).

### Barriers to bringing up sexual problems

**FIG. 3:**
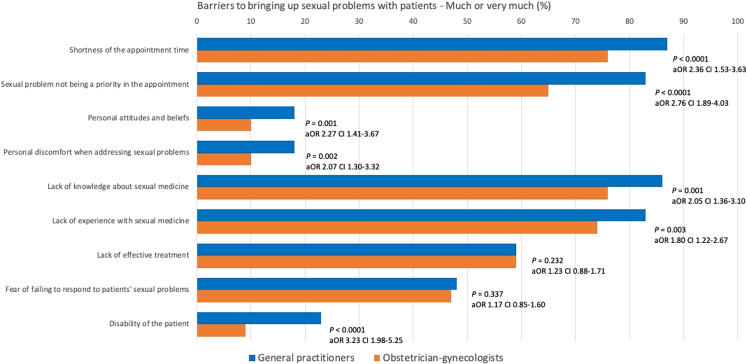
Barriers to bringing up sexual problems with patients. aOR, adjusted odds ratio.

The results of the various barriers to bringing up sexual problems are presented in Figure 3 and Table 3. Compared with OB/GYNs, GPs were more likely to report having barriers in 7 of the 9 categories (“shortness of the appointment time,” ”sexual problem not being a priority in the appointment,” ”personal attitudes and beliefs,” ”personal discomfort when addressing sexual problems,” ”lack of knowledge about sexual medicine,” ”lack of experience with sexual medicine,” and ”disability of the patient”).

**TABLE 3:** Barriers to Bringing up Sexual Problems with Patients

	Much or very much								
	aOR (95% CI)								
	GP (N/total) vs OB/GYN (N/total)								
	Shortness of the appointment time	Sexual problem not being a priority in the appointment	Personal attitudes and beliefs	Personal discomfort when addressing sexual problems	Lack of knowledge about sexual medicine	Lack of experience with sexual medicine	Lack of effective treatment	Fear of failing to respond to patients’ sexual problems	Disability of the patient
Female (*P*)*a*	0.006	<0.0001	0.0001	0.005	0.018	0.026	0.502	0.389	<0.0001
GP vs OB/GYN	1.93 (1.21-3.08)	2.86 (1.89-4.31)	2.69 (1.61-4.48)	1.99 (1.23-3.24)	1.72 (1.10-2.69)	1.63 (1.06-2.49)	1.13 (0.79-1.61)	1.16 (0.83-1.62)	3.17 (1.92-5.25)
	263/298 vs 219/277	248/293 vs 173/266	58/290 vs 25/271	56/297 vs 29/277	254/293 vs 214/275	250/296 vs 209/276	179/280 vs 160/259	155/293 vs 133/275	69/281 vs 24/261
Male (*P*)*a*	0.0004	0.067	0.359	0.272	0.005	0.033	0.102	0.593	0.279
GP vs OB/GYN	7.11 (2.40-21.04)	2.71 (0.93-7.88)	0.54 (0.14-2.03)	3.29 (0.39-27.38)	4.65 (1.60-13.54)	3.05 (1.10-8.46)	2.54 (0.83-7.74)	1.34 (0.46-3.89)	3.20 (0.39-26.34)
	81/99 vs 8/21	75/96 vs 12/20	11/97 vs 4/21	14/99 vs 1/21	82/98 vs 11/21	79/100 vs 11/21	41/94 vs 5/21	33/96 vs 6/21	16/94 vs 1/19
Age <40 (*P*)*b*	0.404	0.003	0.250	0.084	0.866	0.823	0.580	0.031	0.448
GP vs OB/GYN	0.60 (0.18-1.98)	3.24 (1.48-7.11)	1.79 (0.66-4.82)	2.14 (0.90-5.06)	0.92 (0.37-2.32)	0.90 (0.34-2.37)	1.18 (0.65-2.14)	0.53 (0.29-0.94)	1.36 (0.62-2.99)
	134/147 vs 77/81	127/144 vs 60/80	17/145 vs 6/79	25/147 vs 8/82	124/144 vs 73/81	130/146 vs 75/82	78/139 vs 42/74	68/143 vs 54/81	25/135 vs 11/75
Age 40-49 (*P*)*b*	0.123	0.0003	0.039	0.144	0.090	0.102	0.474	0.485	0.041
GP vs OB/GYN	1.86 (0.85-4.10)	3.62 (1.81-7.25)	2.20 (1.04-4.66)	1.83 (0.81-4.12)	1.83 (0.91-3.69)	1.76 (0.89-3.46)	1.26 (0.67-2.35)	1.23 (0.69-2.17)	2.60 (1.04-6.47)
	92/109 vs 85/107	86/106 vs 59/101	25/106 vs 14/106	20/107 vs 12/107	91/108 vs 77/107	87/109 vs 74/105	65/102 vs 64/100	50/108 vs 48/106	20/105 vs 8/101
Age ≥50 (*P*)*b*	<0.0001	0.007	0.014	0.049	0.001	0.004	0.434	0.004	<0.0001
GP vs OB/GYN	4.19 (2.26-7.79)	2.21 (1.24-3.92)	2.76 (1.23-6.20)	2.21 (1.00-4.86)	3.21 (1.66-6.20)	2.33 (1.30-4.17)	1.24 (0.73-2.10)	2.19 (1.29-3.72)	8.13 (3.25-20.30)
	118/141 vs 65/110	110/139 vs 66/105	27/136 vs 9/107	25/142 vs 10/109	121/139 vs 75/108	112/141 vs 71/110	77/133 vs 59/106	70/138 vs 37/109	40/135 vs 6/104

The “cannot say” responses were omitted from the analyses. In each question the responses “much” or “very much” / the number of analyzed responses are shown in the lower column.

aOR >1 indicates that the specific barrier is more likely to be reported as a barrier by the comparison group compared with the reference group.

aOR <1 indicates that the specific barrier is less likely to be reported as a barrier by the comparison group compared with the reference group.

aOR, adjusted odds ratio; GP, general practitioner; OB/GYN, obstetrician-gynecologist.

^*a*^ The multivariable binary logistic regression was carried out with adjustment of age (<40/40-49/≥50 y).

^*b*^ The multivariable binary logistic regression was carried out with adjustment of sex (female/male).

There were interactions between sex and specialty for the barriers of “personal attitudes and beliefs” (*P* = 0.015) and “shortness of the appointment time” (*P* = 0.043). Compared with OB/GYNs, female (but not male) GPs were more likely to report having the barrier of “personal attitudes and beliefs.” Compared with all the OB/GYNs, the GPs of both sexes were more likely to report the barrier of “shortness of the appointment time” (Table 3).

Furthermore, there were interactions between age and specialty regarding the barriers of “shortness of the appointment time” (*P* = 0.014), “lack of knowledge about sexual medicine” (*P* = 0.032), “fear of failing to respond to patients’ sexual problems” (*P* = 0.001), and “disability of the patient” (*P* = 0.026). Compared with OB/GYNs, GPs among the late middle-aged group were more likely to report the barrier of “shortness of the appointment time” and “lack of knowledge about sexual medicine,” but not those between the 2 younger age groups. Compared with OB/GYNs, GPs were more likely to report the barrier of “fear of failing to respond to patients’ sexual problems” among late middle-aged respondents, but not among the middle-aged group. In contrast, compared with young OB/GYNs, young GPs were less likely to report the barrier of “fear of failing to respond to patients’ sexual problems.” Compared with OB/GYNs, middle-aged and late middle-aged GPs were more likely to report the barrier of “disability of the patient,” but not those in the young group (Table 3).

## DISCUSSION

Our study is one of the few to compare GPs’ and OB/GYNs’ attitudes and practice patterns regarding patients’ sexual problems. We found that sexual problems were considered significant clinical issues, as both GPs and OB/GYNs reported that treating sexual problems is an important health care practice. Nevertheless, GPs inquired about sexual problems less frequently and identified more barriers that hindered bringing up patients’ sexual problems than did OB/GYNs. In both younger age groups and among male physicians, these differences in barriers were not equally explicit.

According to our study, OB/GYNs reported inquiring about patients’ sexual problems more often than GPs did. This finding reaffirms a study with reports given by patients.^6^ One explanation for this finding is the different work descriptions of these 2 specialties. The OB/GYN field manages diseases of the female reproductive system and pregnancy, areas in which sexuality is typically highly related. For their part, GPs treat multiple health issues of all specialties, which can be time-consuming. In Finland, GPs also perform basic gynecologic examinations, even though gynecologic problems represent just a fraction of a GP’s work picture. A Norwegian study found that patients brought up an average of 3.3 problems per GP appointment.^25^ Even though in our study, both GPs and OB/GYNs reported that ”shortness of the appointment time” and ”sexual problem not being a priority at the appointment” were barriers to assessment, these barriers were more frequent among GPs. In addition, personal characteristics are often important. In a U.S. study among 248 OB/GYNs and 257 GPs, GPs were more likely to report that ”personal attitudes and beliefs” were barriers to bringing up sexual problems.^16^ We confirmed these results.

Our study supports the importance of education in sexual medicine, as “lack of knowledge” and “lack of experience” were reported as barriers. There are no standards for education in sexual medicine in medical schools.^26^ In a U.S. study among 276 medical trainees, the participants (except for urology and OB/GYN residents) reported feeling unprepared to treat sexual issues.^27^ Similarly, in a Brazilian study of 164 medical students, the teaching of sexual medicine was considered insufficient.^28^ The need to increase education in sexual medicine is also recognized in Finland.^29^ Education in sexual medicine has been shown to enhance physicians’ confidence in managing sexual problems.^16^,^30^,^31^,^32^,^33^ According to a review of 36 articles with global representation regarding sexual health education among health professionals, the lack of standardized sexual health education indicates a gap, raising concerns about students’ proficiency in this area.^34^ Teaching communication skills is also essential for ensuring proper interactions with patients. To improve and standardize education in obstetrics and gynecology, the Nordic Federation of Societies of Obstetrics and Gynecology published an online textbook for medical students.^35^ In addition, the European Sexual Medicine Network plans to prepare sexual medical curricula for university education in Europe (https://www.esmn-cost.eu). Recently a new web-based optional course in sexual medicine was introduced at 2 medical faculties in Finland. In the future, this course will become mandatory for medical students at the University of Turku. Furthermore, follow-up surveys are planned to evaluate the development of education in sexual medicine.

The age of the physician was significant in our study: In the group comparisons between GPs and OB/GYNs, GPs in the oldest age groups were more likely to report difficulty in diagnosing female sexual problems and indicated frequent barriers to bringing up sexual problems. However, between the youngest age groups, few differences were found. In general, over recent decades, the atmosphere around sexuality has become more open-minded and acceptable. Therefore, younger generations may have more skills regarding sexual issues. Overall, access to all information has become easier. A German study with 235 OB/GYNs^2^ and a U.S. study with 1,154 OB/GYNs^17^ showed that younger OB/GYNs asked about patients’ sexual health more often than older ones. The age factor is also found among patients: Younger patients are more likely to report that sexual health is an important part of general well-being^6,7^ and that health care providers should frequently ask their patients about sexual health.^6^

### Strengths and limitations

The strength of our study was the use of web-based questionnaires, which permitted anonymity. The questionnaires were programmed to not proceed if any response was missing, so all returned questionnaires were complete. This provided comprehensive data but could have lowered the response rate, as some potential participants could have considered answering too time-consuming. However, the OB/GYN respondents represented one-third of the specialists in obstetrics-gynecology in Finland,^22^ and of enrolled GPs, almost half responded. Both data sets were comparable, as the age distributions were similar. Sex proportions were slightly different among the groups, but they corresponded to the sex proportions of physicians in Finland.^21,22^ Nevertheless, the proportion of male OB/GYNs was small, so findings regarding sex differences should be confirmed using larger samples.

No statistics about nonrespondents were available for comparison, as we had no access to the Finnish Medical Association or the Finnish Society of Obstetrics and Gynecology registries. Furthermore, our study included only Finnish GPs and OB/GYNs; therefore, our results might not be directly applicable to physicians in other countries. In addition, health care systems differ between countries, which can have an effect on outcomes. Our results were self-reported, so the actual management of patients’ sexual problems was not measured.

## CONCLUSION

According to our study, both GPs and OB/GYNs considered the treatment of sexual problems to be important in health care. Although several barriers arose among both specialties, GPs were more likely to report barriers and inquire about sexual problems less regularly. Continuing education is warranted to improve the engagement with patients’ sexual problems.
